# Learning approaches and their association with academic performance among undergraduate students in a dental teaching institution in India

**DOI:** 10.3389/fdmed.2026.1716151

**Published:** 2026-04-23

**Authors:** Venkitachalam Ramanarayanan, Parvathy Balachandran, Anju James, Gowri A. Anu, Joe Joseph, Beena Varma

**Affiliations:** 1 Department of Public Health Dentistry, Amrita Vishwa Vidyapeetham Amrita School of Dentistry, Kochi, India; 2 Department of Public Health Dentistry, Sree Mookambika Institute of Dental Sciences, Kanyakumari, India; 3 Department of Oral Medicine and Radiology, Amrita Vishwa Vidyapeetham Amrita School of Dentistry, Kochi, India

**Keywords:** academic performance, dental education, dental students, learning, learning preferences

## Abstract

**Purpose:**

This cross-sectional survey was aimed to assess the preference of learning approach and its correlation with academic performance in a dental teaching institution.

**Methods:**

The survey was conducted in a dental teaching institution among all undergraduate students using Revised Two-Factor Study Process Questionnaire (R-SPQ-2F).

**Results:**

A total of 155 students participated in the study. A statistically significant positive correlation between deep approach scores and academic performance (*r* = 0.212, *p* = 0.008) was obtained indicating that students who adopted deeper learning approach tended to perform better academically. Across the years of study, mean deep approach scores were higher than mean surface approach scores. This difference was statistically significant for the Third year (*p* = 0.007), Final year (*p* = 0.001), Internship (*p* < 0.001) and the combined clinical group (*p* < 0.001).

**Conclusion:**

The mean scores of deep and surface approaches (and its sub-domains) increased over the years in the pre-clinical and clinical phase.

## Introduction

Learning represents a dynamic interplay between behavioral changes and environmental stimuli, culminating in knowledge acquisition in an institutional context (1, 2). Over the years, learning has gained growing interest in education research yielding valuable insights into concepts of learning styles and approaches. These provided robust theoretical frameworks to understand ways in which an individual perceived, processed, and assimilated knowledge.

Learning styles broadly refers to methods like reading, group discussions, or lectures that learners use to acquire knowledge. It is the “characteristic cognitive, effective, and psychosocial behaviors that serve as relatively stable indicators of how learners perceive, interact with, and respond to the learning environment” (3). Learning approaches, on the other hand, refers to changes in a learner's intentions, behaviour and study habits according to their perceptions of a learning task (4). An individual with deep approach to learning is guided by an intrinsic motivation to acquire knowledge while an individual of surface approach category is motivated by events like examinations (5). Both these concepts have been utilized in educational research to enhance learning outcomes. However, this has limitations like the failure to explain the underlying cognitive mechanism of an individual's learning style, and use of less valid measures (6). Studies also criticize that exclusive focus on learning styles cannot improve learning outcomes (6, 7). This underscores the need for a more dynamic concept such as learning approaches to enhance academic outcomes.

Based on motivation, learning approaches are categorized as deep and surface (8). Curry's Onion model of learning behaviors further suggests that learning approaches offer a better avenue for enhancing learning outcomes than learning styles. According to this model, learning behavior is multi-layered like an onion, with the outermost layer denoting instructional preferences, information processing style in the middle layer, and the innermost layer comprising cognitive personality style (9, 10). The degree of flexibility to interventions increases from innermost to outer layers. Therefore, to effectively improve learning outcomes, educational interventions should target the layer most responsive to changes—instructional preferences (9). This highlights the importance of learning approaches because, unlike learning styles in the less modifiable middle layer, they occupy the more flexible outer layer. Targeting this window of opportunity offered by learning approaches is particularly important in health professions education where students engage with both theoretical and clinical experiences. Health professions curriculum demands critical thinking, professional-judgement and insightful learning that can be achieved through deep learning. Therefore, assessment of learning approaches among students is a prerequisite to improve their academic performance.

The relationship between learning approaches and academic performance in health professions education has been consistently demonstrated across multiple studies (11–13). Though learning styles and preferences in dental education have been studied using various instruments like VARK scale, research to assess learning approaches and their association with academic performance especially in the Indian context is notably lacking. The Revised Two factor Study Process Questionnaire (R-SPQ-2F) is a tool used to assess the learning by categorizing it into deep and surface approaches. The “deep approach” encompasses deep motive (intrinsic interest in learning) and deep strategy (relating ideas and seeking meaning), while the “surface approach” includes surface motive (fear of failure and external pressure) and surface strategy (rote learning and memorization) (14). This two-factor structure has been validated across diverse educational contexts and cultural settings, demonstrating the instrument's robustness and applicability in various higher education environments. Its ease of use and short length further strengthen its advantages over other assessment tools (13). Dental education being predominantly a skill-based course, this assessment is particularly relevant as the R-SPQ-2F is a psychometrically validated tool to distinguish between deep and surface learners (5). In the Indian context, a study among medical students during distance learning using the Revised Two-Factor Study Process Questionnaire (R-SPQ-2F) showed a gender based predilection for learning approaches with females preferring deep learning approaches than their male counterparts (15). However, this study was limited to distance learning setting and did not address the distinct curricular demands and substantial clinical exposure of conventional medical/dental education. Therefore, there is a critical need for a methodologically robust study to determine the association between the learning approaches and academic performance.

Thus, the present study was designed was to assess the association between learning approaches and academic performance among dental undergraduate students in a teaching hospital in India.

## Methodology

### Study design

A cross-sectional study was conducted to assess the learning style preferences among undergraduate dental students and to explore the relationship between learning styles and academic performance.

### Study setting

The study was conducted at a dental teaching hospital in the state of Kerala, India.

### Selection of study participants and sample size

#### Inclusion criteria

This was a census-type survey conducted among the undergraduate students at a dental teaching hospital in India. All undergraduate students enrolled in the BDS program in the institution during the study period were eligible to participate. This method was chosen to ensure maximum coverage and to minimize the risk of sampling bias. Participation was entirely voluntary, and students were given adequate time and flexibility to complete the survey. As such, *a priori* sample size estimation or power calculation was not performed. The final sample size was determined by the number of eligible participants available during the study period (*n* = 240) and their willingness to participate.

#### Exclusion criteria

Students who were not willing to participate or who did not provide written informed consent were excluded from the study.

### Study tool

The study employed the R-SPQ-2F questionnaire consisting of 20 items designed to assess two primary learning approaches: deep approach and surface approach, each comprising both motivational and strategic components divided equally. Each item is rated on a 5-point Likert scale ranging from “never or only rarely true of me” to “always or almost always true of me”.

### Conduct of the study

Students were approached during academic hours and were briefed about the study's objectives and procedures. Those who expressed willingness to participate were enrolled in the study after obtaining their informed and written consent.

The questionnaires were self-administered through a secure online survey platform. The survey was conducted in the presence of the investigators to clarify any doubts and ensure completeness of responses.

Students' academic performance was assessed by collecting their scores (marks) obtained in the most recently completed summative university examination. These academic scores were used to analyse correlations between specific learning style approach and academic achievement. Students were also categorized into preclinical (1st and 2nd year BDS) and clinical groups (3rd, 4th year BDS and internship) for analysis.

### Statistical analysis

Analysis was done using IBM Statistical Package for the Social Sciences (SPSS) Statistics for Windows, version 20 (IBM Corp., Armonk, N.Y., USA). Learning approach domain and sub-domain scores were presented as mean and standard deviation. Comparison of domain and sub-domain scores with year of study was done using One-way ANOVA. Within group analysis was done using unpaired and paired t-test. A *p*-value of less than 0.05 was considered statistically significant.

### Ethical considerations

The study received approval from the Institutional Ethics Committee (IEC) of Amrita Institute of Medical Sciences, Kochi (ECASM-AIMS-2024-097 dated 20-02-2024). Online informed consent was obtained from each participant prior to inclusion in the study. The anonymity and confidentiality of all participants were strictly maintained throughout the research process and stated explicitly in the participation information sheet. All survey responses were collected without personally identifiable information and were accessible only to the research team. Academic performance data were linked to survey responses using anonymized codes to protect participant identities. Data were analyzed and reported only in aggregate form to further ensure participant confidentiality. Participation in the study had no bearing on students' academic standing or evaluation.

## Results

**Figure 1:**
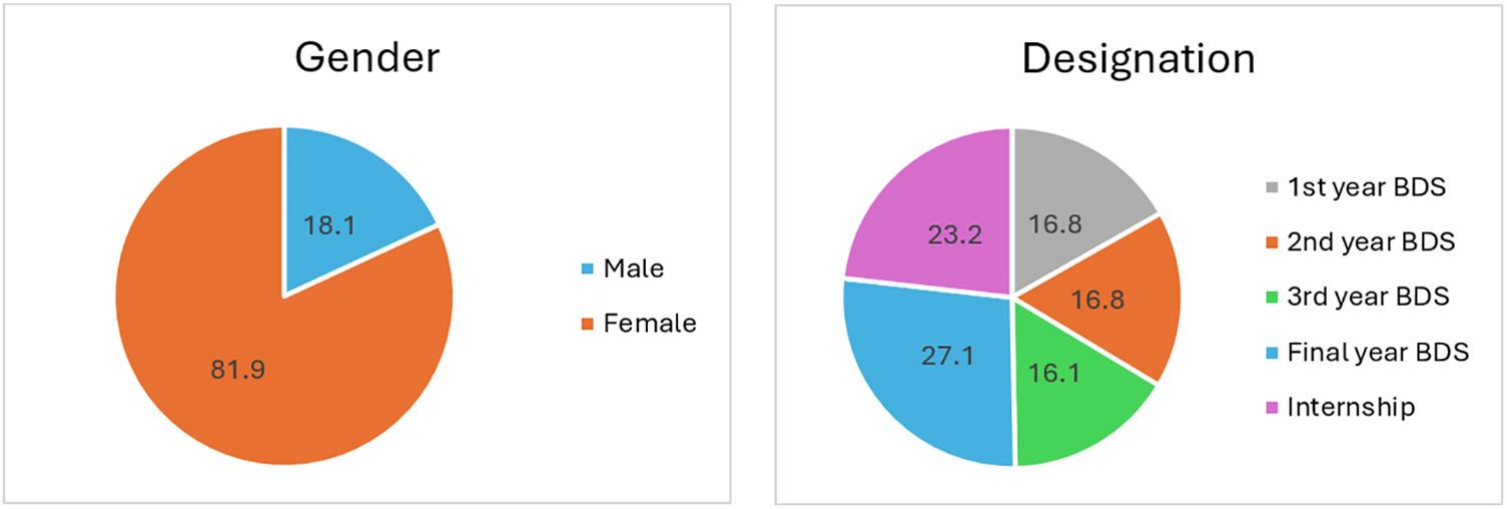
Demographic characteristics of study participants.

A total of 155 undergraduate dental students participated in the study, yielding a response rate of approximately 65%. Among the participants, the majority were female (81.9%, *n* = 127), with males accounting for 18.1% (*n* = 28). Students from all academic years of the BDS program were represented: First Year (16.8%), Second Year (16.8%), Third Year (16.1%), Final Year (27.1%), and Internship (23.2%) (Figure 1).

The Revised Two-Factor Study Process Questionnaire (R-SPQ-2F) scores were used to assess students' learning styles. The mean scores for the learning subscales were as follows: deep motive—13.01 ± 3.51, deep strategy—13.08 ± 3.67, surface motive—10.35 ± 3.66, and surface strategy—12.40 ± 3.96. The composite scores for deep approach and surface approach were 26.09 ± 6.83 and 22.75 ± 6.95, respectively (Table 1).

**Table 1:** Mean domains and sub-domain scores of of R-SPQ-2F questionnaire (*n* = 155).

Domain/Sub-domain	Mean ± SD
Deep Motive	13.01 ± 3.51
Deep Strategy	13.08 ± 3.66
Surface Motive	10.35 ± 3.66
Surface Strategy	12.40 ± 3.95
Deep Approach	26.09 ± 6.83
Surface Approach	22.75 ± 6.95

Comparison of mean scores of learning approach scales and sub-scales across the academic years revealed that, deep approach (the combined measure of deep motive and strategy), was highest among second year (27.77) and internship students (27.03), and lowest in first year (24.96) and third year (24.84) students. The differences were not statistically significant (*p* = 0.418). Similarly for surface approach, (the combined measure surface motive and strategy), showed minor differences across years, highest in second year (25.31) and least in third year (21.04), though this was not statistically significant (*p* = 0.236) (Table 2).

**Table 2:** Comparison of mean domain and sub-domain scores of R-SPQ-2F questionnaire across study years.

Domain/Sub-domain	First year BDS	Second year BDS	Third year BDS	Final year BDS	Internship	*p* value
Deep Motive	12.58 ± 4.13	13.73 ± 4.03	12.52 ± 2.63	12.93 ± 3.25	13.22 ± 3.53	0.716
Deep Strategy	12.38 ± 4.56	14.04 ± 3.78	12.32 ± 2.21	12.76 ± 3.16	13.81 ± 4.11	0.240
Deep Approach	24.96 ± 8.48	27.77 ± 7.49	24.84 ± 4.30	25.69 ± 5.96	27.03 ± 7.37	0.418
Surface Motive	10.54 ± 4.09	10.88 ± 4.23	9.68 ± 2.91	10.40 ± 3.89	10.22 ± 3.18	0.829
Surface Strategy	12.46 ± 4.91	14.42 ± 3.94	11.36 ± 3.05	12.31 ± 3.61	11.72 ± 3.80	0.045*
Surface Approach	23.00 ± 8.66	25.31 ± 7.01	21.04 ± 5.46	22.71 ± 6.79	21.94 ± 6.46	0.236

* *p* value significant at less than 0.05.

**Figure 2:**
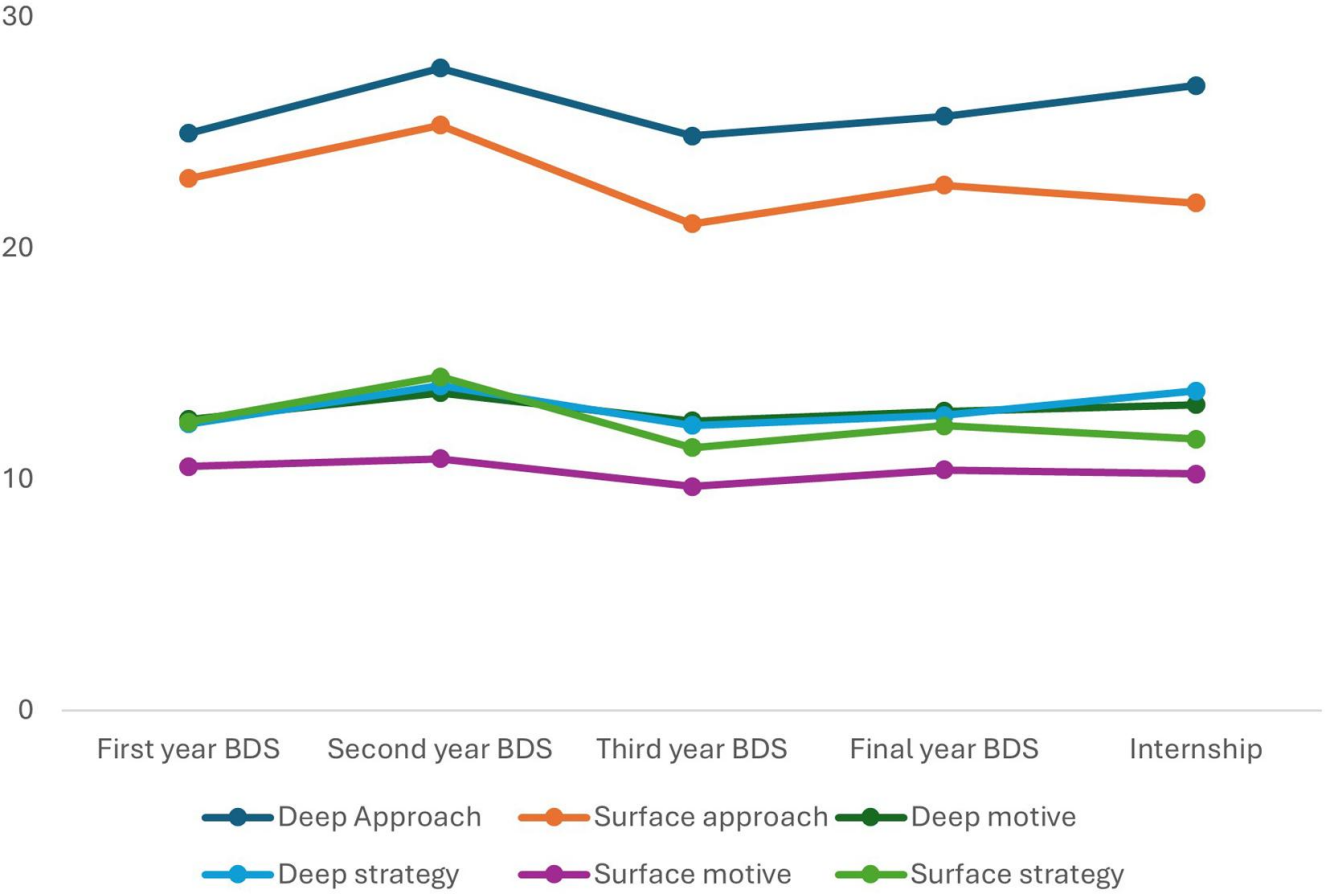
Comparison of mean domain scores of R-SPQ-2F questionnaire across study years.

Deep motive scores were relatively consistent across all academic years, ranging from 12.52 (third year) to 13.73 (second year), with no statistically significant differences (*p* = 0.716) while deep strategy scores showed a slight increase from first year (12.38) to internship (13.81), However, this observation was also not statistically significant (*p* = 0.240). Surface motive scores were uniform across all years, with the highest mean in second year (10.88) and lowest in third year (9.68) and did not show any significant variation (*p* = 0.829) while surface strategy scores varied more notably, with second year students showing the highest mean (14.42) and third year students the lowest (11.36). The difference across years was statistically significant (*p* = 0.045) (Table 2, Figure 2). Scores across all domains and subdomains increased from the first to the second year, declined in the third year, and then increased again in the final year. However, these variations were not statistically significant, except for the scores related to the surface learning strategy.

On comparison of deep and surface approach scores within each year of study and combining the preclinical and clinical students, it was observed that mean scores of deep approach were higher than surface approach for all groups (Figure 2). The difference was statistically significant for the Third year (*p* = 0.007), Final year (*p* = 0.001), Internship (*p* < 0.001) and the combined clinical group (*p* < 0.001) (Table 3).

**Table 3:** Comparison of learning approach scores among preclinical and clinical students and year of study.

Designation	Learning approach	Mean ± SD	*p* values	95% CI
First Year BDS (*n* = 26)	Deep Approach	24.96 ± 8.48	0.339	−2.180–6.103
	Surface Approach	23.00 ± 8.66		
Second Year BDS (*n* = 26)	Deep Approach	27.77 ± 7.49	0.214	−1.516–6.439
	Surface Approach	25.31 ± 7.01		
Third Year BDS (*n* = 25)	Deep Approach	24.84 ± 4.30	0.007*	1.122–6.478
	Surface Approach	21.04 ± 5.46		
Fourth Year BDS (*n* = 42)	Deep Approach	25.69 ± 5.96	0.001*	1.308–4.644
	Surface Approach	22.71 ± 6.79		
Internship (*n* = 36)	Deep Approach	27.03 ± 7.37	<0.001*	2.611–7.556
	Surface Approach	21.94 ± 6.46		
Preclinical (*n* = 52)	Deep Approach	26.37 ± 8.04	0.115	−0.561–4.984
	Surface Approach	24.15 ± 7.89		
Clinical (*n* = 103)	Deep Approach	25.95 ± 6.17	<0.001*	2.667–5.158
	Surface Approach	22.04 ± 6.35		

* *p* value significant at less than 0.05.

A Pearson correlation analysis revealed a statistically significant weak positive correlation between deep approach scores and academic performance (*r* = 0.212, *p* = 0.008), indicating that students who adopted deeper learning strategies tended to perform marginally better academically. In contrast, no significant correlation was found between surface approach scores and academic performance (*r* = −0.035, *p* = 0.669) (Table 4).

**Table 4:** Correlation between learning approach scores and summative marks.

	Summative exam marks	
Learning approach	Pearson Correlation Coeff.	*p* value
Deep Approach (*N* = 155)	0.212	0.008*
Surface Approach (*N* = 155)	−0.035	0.669

* *p* value significant at less than 0.05.

## Discussion

Teaching strategies must be adapted to the Learning approach of the respective courses in which teaching is carried out (16). Understanding Learning approach help teachers to develop multimodal learning approaches more than individualized approaches to encourage deeper learning in students (17). In recent years, dental education has witnessed significant advance-ments and curriculum shifts in response to evolving healthcare needs, techno-logical advances, and changing societal demands (18). But little attention is given to assess the effect of these activities on the learning process of the students. Identifying prevailing learning approaches allows educators to tailor strategies that promote deeper engagement and long-term retention of knowledge. In this study, the Revised Two-Factor Study Process Questionnaire (R-SPQ2F) was used as a validated tool to assess the deep and surface learning approaches among undergraduate dental students.

### Learning style (deep approach/surface approach) across academic years

All students showed a preference for deep approach over the surface approach. This observation was significant in the clinical years (Third year, Final year and Internship) of the course. This trend may be attributed to the fact that there is an increased exposure to clinical practice (patients) requiring application of practical knowledge, compared to more theoretical knowledge in the pre-clinical years (19). These results were consistent with the studies by Alahmari et al. 2023 (13), Rabi et al. 2024 (19), Haghparast et al. (20) and, Kiran and Kulsoom 2019 (21) which reported that senior students tend to adopt deeper learning strategies.

First-year students in our study recorded lower deep approach scores. This could probably be due to reliance on memorization-oriented learning habits carried over from their secondary school. Introduction of unfamiliar medical and dental terminologies pose an additional challenge, as students must expand their vocabulary while adjusting to the demands of professional education. As a result, they adopt a surface approach by memorizing the facts and using mnemonics to recall information in their initial years of undergraduate education.

A decline in deep approach scores among third-year students could be linked to the stress associated with transitioning to clinical training, as has been reported in previous literature (22). Transitioning to clinical years has been studies in various contexts. While the students have acknowledged that the shift is challenging, it could be overcome through strategies like mentoring from seniors, early and robust orientation sessions and stress management programs (23–25). At internship, there is a drop in the surface approach scores. These results are consistent with those of other studies that show a drop in surface approaches with time spent in dental schools (26). An increase in both surface and deep approaches in the second year were observed. The increase in surface approach may be linked to reduced intrinsic motivation and a heightened fear of examination failure (27). These patterns align with the observations of Premkumar et al. (28), where heavy workloads and assessments emphasizing memorization can discourage self-directed learning, pushing students toward surface strategies. Students' perception of heavy workload and inappropriate assessment persuade them toward a surface approach, while perceptions of good teaching persuade them toward deep approaches to study (29). Obtaining the feedback from students about the design and implementation of the learning environment will help educators identify what has worked and where improvements could be made in the future (30).

The study findings are supported by robust theoretical frameworks of self-regulated learning theory and Bigg's 3P Model. In Zimmerman's self-regulated learning theory, the learner proactively evolves through the cyclic phases of forethought, performance, and self-reflection (31). The phases of forethought and self-reflection closely align with the goal- oriented learning of deep learning approach and the resultant academic performance resonates with the performance phase. Therefore, as the student progress into clinical years, the increased self-regulation involving these underlying psychological mechanisms promotes deep learning. This explains the higher mean scores of deep approaches in the clinical years like third year, Final year and Internship. This increase in deep learning approach as the student moves from the preclinical first and second years to clinical senior years and internship is further supported by the Biggs 3P Model (32). According to this model, the student's transition from preclinical to clinical training is the presage factor that promotes the process of deep learning resulting in better academic performance—the product.

### Association between learning approaches and academic performance

A significant positive correlation was observed between deep learning approaches and academic performance, indicating that students adopting deep strategies tended to achieve higher examination scores. This finding is in agreement Tarek et al. (2023) (19), who reported that students adopting a deep approach attained higher scores (above 70%). Few studies which measured the scores in Grade Point Average (GPA) also showed an association with the learning strategy (13, 33, 34). This shows that more deep approach learning strategies can provide better academic performance due to a more application-based approach of learning (35–37). However, Wilkison *et al*. (38) concluded that learning approach does not affect students' academic performance.

### Implications for dental education

Our results highlight the need to integrate deep approach–promoting strategies across all stages of dental education, particularly in the early years, where surface learning tendencies are more prevalent. This provides a need for more application-based learning approaches, like problem-based learning and flipped classrooms, which are limited in the early years of current dental training. Additionally, implementing problem-based learning through vertically and horizontally integrated curricula will help students improve their critical thinking and problem-solving, so they can adopt a deep approach. Pre and post-test study can be carried out to measure the learning approaches in specific subjects with the help of single teaching strategy i.e., Problem Based Learning and type of assessment; as assessment drives student learning (39). Assessment enables us to measure timely outcomes and changes in learning approaches.

Since this study was conducted in a single institution, the findings may primarily reflect the context and characteristics of this setting. However, dental curricula and teaching methods are largely uniform across the country. As it was a cross-sectional study, the correlation with academic performance was measured based on the scores (marks) conducted in the last concluded final summative examination which would not have been ideal. We achieved a response rate of 65%, which may be attributed to the voluntary nature of participation and could have introduced the possibility of non-response bias. For a more deeper understanding of the changes in learning approach, further studies could focus on following up the students through out the course.

## Conclusion

This study shows that the scores of all domain and sub-domains of R-SPQ-2F showed an increase in the first two years followed by a drop in third year BDS and subsequent increase in the succeeding years highlighting the transition from pre-clinical to clinical education. Deep approach showed a weak positive correlation with academic outcome.

## Data Availability

The raw data supporting the conclusions of this article will be made available by the authors, without undue reservation.
